# Exegesis on practice-changing evidence for low-risk differentiated thyroid carcinoma in thyroidology: ab ovo usque ad mala?

**DOI:** 10.1590/1806-9282.20252240

**Published:** 2026-10-12

**Authors:** Ilker Sengul, Demet Sengul

**Affiliations:** 1 Giresun University, Faculty of Medicine, Thyroidology Unit – Giresun, Turkey.; 2 Giresun University, Faculty of Medicine, Division of Endocrine Surgery – Giresun, Turkey.; 3 Giresun University, Faculty of Medicine, Department of General Surgery – Giresun, Turkey.; 4 Giresun University, Faculty of Medicine, Department of Pathology – Giresun, Turkey.

Dear Editor,

*Ad augusta per angusta!* We have perused with palpable interest the illuminating comment penned by Hartl^1^, bearing the title "Practice-changing evidence for low-risk differentiated thyroid cancer" in thyroidology^1-6^, published in volume 406, *The Lancet*, which discusses the salient findings emerging from the pivotal IoN trial, led by Mallick et al.^2^, published in the same volume of *The Lancet*. The escalating global incidence of thyroid carcinoma, with the preponderance of early-stage presentation being observed within high-income nations, renders the critical debate concerning potential overtreatment a matter of profound clinical and economic moment. As such, Hartl's^1^ analysis rightly emphasizes the dramatic paradigm shift now underway in low-risk disease, characterized by tumors of stage T1 or T2, with nodal status N0 or Nx, where the recurrence rate is estimated at under 5% and disease-specific mortality remains below 1%. The IoN trial, a substantial prospective, randomized investigation including 504 patients, provides irrefutable, high-level confirmation that the post-operative omission of radioiodine ablation is demonstrably non-inferior to systematic treatment for this cohort, which finding significantly buttresses the conclusions drawn from the preceding ESTIMABL2 trial. The measured Kaplan-Meier 5-year estimated disease-free survival rate for the cases spared ablation was recorded at 97.9% (96.1–99.7), which compares most favorably with the 96.3% (93.9–98.7) observed in the ablation arm. Notably, no fatalities attributable to thyroid cancer were recorded across either cohort. Furthermore, the economic ramifications are compelling, suggesting a potential saving of 100 per patient within the UK's National Health Service, solely related to the costs of hospitalization and radioiodine administration for these procedures. Of the 504 patients randomly assigned, 251 were allocated to the no ablation group. Moreover, it remains incumbent upon the academic community to reflect upon the precise nuances elaborated on by Hartl^1^, particularly regarding the broadened inclusion criteria of the IoN study. The trial admitted a minority of patients exhibiting T3 or T3a status (46 patients, or 9%) and N1a tumors (47 patients, or 9%). While this expansion beyond the confines of ESTIMABL2 is scientifically notable, the authors of the original Comment wisely caution against applying these definitive results to this intermediate subgroup, owing to the trial's lack of statistical power for rigorous subgroup analysis. Of note, despite the potential cost savings identified, a comprehensive, accurate cost-effectiveness analysis encompassing the entirety of the patient pathway remains unperformed. Likewise, the IoN trial was not designed to permit definitive conclusions regarding the predictive value of postoperative thyroglobulin concentrations for recurrence, meaning no such assertions may be drawn. The collective evidence, now profoundly reinforced by the IoN outcomes, serves as a crucial determinant in advancing the necessary individualization of therapy and mitigating the endemic issue of overtreatment in low-risk differentiated thyroid carcinoma. The logical progression, as articulated in the comment, suggests that proposing thyroid lobectomy for patients presenting with unilateral tumors may now be considered prudent. However, this shift mandates validating new surveillance algorithms, given the known unreliability of serum thyroglobulin as a predictor of tumor recurrence following lobectomy. Ultimately, a superior delineation of the underlying molecular architecture of these cancers is indispensable for reliably forecasting progression and recurrence, thereby ensuring that therapeutic intervention is reserved solely for those patients genuinely requiring it. This body of research provides a significant and necessary advancement in the clinical delineation of treatment pathways for differentiated thyroid cancer. This issue merits further investigation. We extend our sincere felicitation to Hartl^1^ for their scholarly work in *The Lancet* on low-risk differentiated thyroid carcinoma for thyroidologists. *Felix qui potuit rerum cognoscere causas*.

## Data Availability

The datasets generated and/or analyzed during the current study are available from the corresponding author upon reasonable request.
